# Orthopaedic, trauma surgery, and Covid-2019 pandemic: clinical panorama and future prospective in Europe

**DOI:** 10.1007/s00068-022-01978-z

**Published:** 2022-05-06

**Authors:** Filippo Migliorini, Christian David Weber, Geatano Pappalardo, Hanno Schenker, Ulf Krister Hofmann, Joerg Eschweiler, Frank Hildebrand

**Affiliations:** 1 Department of Orthopaedic, Trauma, and Reconstructive Surgery, RWTH University Hospital, Pauwelsstraße 30, 52074 Aachen, Germany; 2 Department of Orthopaedic Surgery, Klinikum Ernst Von Bergmann, Potsdam, Germany

**Keywords:** Covid, Pandemic, Orthopedics, Trauma, Sports medicine, Europe

## Abstract

**Purpose:**

This study investigated the impact of the Covid-19 pandemic in Europe on consultations, surgeries, and traumas in the field of orthopaedic and trauma surgery. Strategies to resume the clinical activities were also discussed.

**Methods:**

This systematic review was conducted according to the Preferred Reporting Items for Systematic Reviews and Meta-Analyses: the 2020 PRISMA statement. All the comparative studies reporting data on the impact of Covid-19 in the field of orthopaedic and trauma surgery in Europe were accessed. Only comparative clinical studies which investigated the year 2020 versus 2019 were eligible.

**Results:**

57 clinical investigations were included in the present study. Eight studies reported a reduction of the orthopaedic consultations, which decreased between 20.9 and 90.1%. Seven studies reported the number of emergency and trauma consultations, which were decreased between 37.7 and 74.2%. Fifteen studies reported information with regard to the reasons for orthopaedic and trauma admissions. The number of polytraumas decreased between 5.6 and 77.1%, fractures between 3.9 and 63.1%. Traffic accidents admissions dropped by up to 88.9%, and sports-related injuries dropped in a range of 59.3% to 100%. The overall reduction of the surgical interventions ranged from 5.4 to 88.8%.

**Conclusion:**

The overall trend of consultations, surgeries, and rate of traumas and fragility fractures appear to decrease during the 2020 European COVID pandemic compared to the pre-pandemic era. Given the heterogeneities in the clinical evidence, results from the present study should be considered carefully.

**Level of evidence:**

Level IV, systematic review.

## Introduction

The SARS-CoV-2 pandemic made clear that the established administrative structures of the health authorities are generally not prepared to face the immediate challenges of such a major infectious risk or permanent crisis situation. Many national health authorities acted independently and adapted their strategies, evidencing that the countries of the European Union were not able to reach a collective consensus and to adopt common guidelines. The pandemic caused by Covid-19 has been confronting the healthcare landscape with new challenges [1, 2]. In addition to the already highly burdened hospitals, the pandemic management required further considerable organizational efforts [3–5]. A high degree of flexibility and willingness to improvise for clinical employees and staff responsible for the organization were required [6–8]. Often far-reaching process changes, such as the management of patient flows, had to be implemented in everyday clinical practice within a very short period of time [9].

Though orthopaedic and trauma surgery are not disciplines directly involved in the clinical management of Covid-19 patients, the pandemic caused profound changes in patient flow management, impacting the clinical practice and requiring significant management efforts from medical and non-medical personnel [10–12]. This study investigated the impact of the Covid-19 pandemic in Europe on consultations, surgeries, and traumas in the field of orthopaedic and trauma surgery. Strategies to resume the clinical activities were also discussed.

## Methods

### Eligibility criteria

All clinical investigations reporting data on the impact of Covid-19 in the field of orthopaedic and trauma surgery in Europe were accessed. Studies focusing on consultations and surgeries, sports medicine, fragility fractures, and European trauma registries were included. Study level I to III of evidence, according to Oxford Centre of Evidence-Based Medicine [13], were considered. Given the authors language capabilities, articles in English, German, Italian, French and Spanish were eligible. Comparative studies published in peer reviewed journals were considered. Studies published in grey literature or without full-text were not eligible. Studies which have been conducted in other continents rather than Europe were not suitable. Only comparative studies which investigated the year 2020 *versus* the pre-COVID era were suitable. Reviews, editorials, comments, and expert opinions were excluded.

### Search strategy

This systematic review was conducted according to the Preferred Reporting Items for Systematic Reviews and Meta-Analyses: the 2020 PRISMA statement [14]. The PICOT algorithm was preliminary pointed out to guide the search:

- P (Population): Orthopaedic and Trauma patients;
- I (Intervention): Patient management;
- C (Comparison): 2020 versus 2019;
- O (Outcomes): consultations and surgeries, sports medicine, fragility fractures, trauma registries;
- S (Source): European Orthopaedic and Trauma centres
- T (Type of study): clinical investigation.

In December 2021, the following databases were accessed: Pubmed, Web of Science, Google Scholar, Embase, with no time constrains. The following keywords were used in combination using the Boolean operators AND/OR: *Covid, Sars, 2019, 2020, pandemic, Coronavirus, Europe, European, orthopaedic, trauma, traumatology, surgeries, intervention, management, treatment, surgical, consultations, surgeries, sport medicine, fragility fractures, registries*.

### Selection and data collection

Three authors (FM; HS; GP) independently performed selection and data collection. All the resulting titles were screened and if suitable, the abstract was accessed. The full-text of the abstracts which matched the topic were accessed. A cross reference of the bibliography of the full-text articles was also performed. Disagreements were debated and the final decision was made by the main author (FM).

## Results

### Search results

**Fig. 1:**
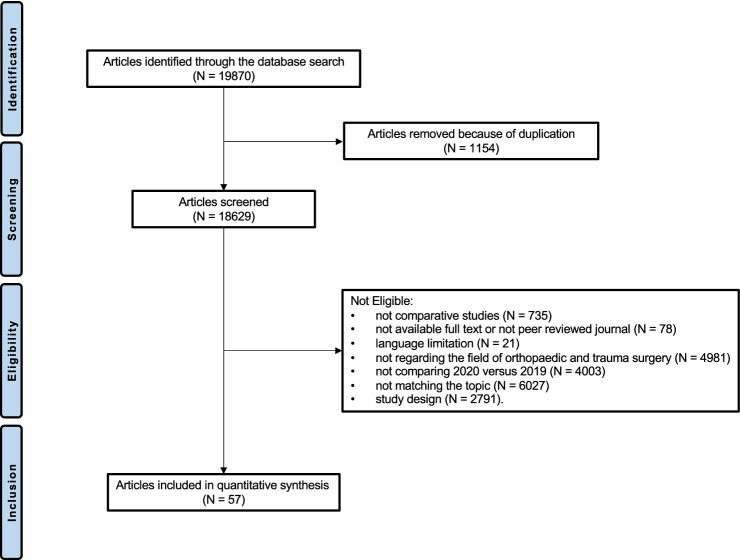
Flow-chart of the literature search

The initial literature search resulted in 19,870 articles. 1154 studies were removed as they were duplicates. Further 18,629 studies were excluded as they did not match the eligibility criteria: not comparative studies (*N* = 754), not available full text or not published in peer reviewed journals (*N* = 78), language limitations (*N* = 21), not in the field of orthopaedic and trauma surgery (*N* = 4981), not comparing 2020 versus 2019 (*N* = 4003), not matching the topic (*N* = 6027), study design (*N* = 2791). Finally, 57 clinical investigations were included (Fig. 1).

### Results syntheses

Elective surgeries and nonurgent consultations were deferred to reduce people contacts [15, 16]. Fourteen studies reported information with regard to the number of consultations [15–28]. Eight studies reported a reduction of the orthopaedic consultations, which decreased between 20.9 and 90.1% [17–19, 22, 23, 26, 27, 29]. Seven studies reported the number of emergency and trauma consultations, which were decreased between 37.7 and 74.2% [15, 16, 21, 22, 25, 27, 28]. Fifteen studies reported information with regard to the reasons for orthopaedic and trauma admissions [15–17, 19, 21, 23, 26, 28–34]. The number of polytraumas decreased between 5.6 and 77.1%, fractures between 3.9 and 63.1% [21, 23, 34]. Traffic accidents admissions dropped by up to 88.9%, and sports related injuries dropped in a range of 59.3–100% [15, 33]. Domestic injuries dropped between 20 and 50% in five studies [15, 23, 29], while an increased trend ranging between 122 and 300% was evidenced in three studies [17, 30, 33]. Seventeen studies reported information on the number of orthopaedic and trauma surgical interventions [15, 17–23, 30–38]. The overall reduction of the surgical interventions ranged from 5.4 to 88.8%. Only one study reported an increase of 47.8% [32]. The overall number of surgeries performed in elective regimes decreased between 33.3 and 100% [15, 18, 20, 23, 33, 37–39]. Unplanned surgical interventions due to traumas decreased in a range from 21.2 to 66.7% [15, 17, 20, 21, 23, 30, 34, 37], while three authors evidenced an increase between 32.1% and 94.2% [33, 35, 38]. The studies which reported data on consultations and surgeries during the year 2020 versus the pre-Covid-19 era in Europe are shown in Table 1.

**Table 1:** Studies which reported data on consultations and surgeries during the year 2020 versus the pre-Covid-19 era (ORCA: Orthopaedic Research Collaborative East Anglia)

Author, year	Journal	Design	Country	Main findings
Andreata et al., 2020 [35]	*Int Orthop*	Retrospective	Italy	These data clearly show the deep impact of the Covid-19 pandemic on OR facilities. Efficiency indicators fell dramatically in April 2020 compared with the corresponding period in 2019. This scenario will deeply affect both the waiting lists and the economic burden of the hospital. Regaining efficiency and maintaining the quality and safety of the process while restoring elective orthopaedic surgery are among the main challenges that surgeons will face in the next time
Benazzo et al., 2020 [15]	*Int Orthop*	Observational	Italy	Covid-19 outbreak showed a tremendous impact on all orthopaedic trauma activities throughout the country except for the surgical treatment of femoral neck fractures, which, although reduced, did not change in percentage within the analyzed timeframe
Druel et al., 2020 [30]	*Int Orthop*	Retrospective	France	Containment measures had a direct impact on trauma surgery activity with a decrease of a third of trauma surgery activity. Those results could be useful if a new pandemic occurred
Ghermandi et al., 2020 [32]	*Eur Rev Med Pharmacol Sci*	Retrospective Observational	Italy	Surgical activity was paradoxically increased during SARS-CoV-2 pandemic lockdown through the management of urgent and non-deferrable spinal disease with a low rate (3,9%) of Covid-19 infections
Giuntoli et al., 2020 [23]	*J Clin Orthop Trauma*	Retrospective	Italy	The Covid-19 pandemic raised many important issues, such as the optimal management of patients requiring the treatment of conventional diseases during a pandemic. The flow of patients changes from one area to another during a pandemic and an integrated approach within the same geographical area could be useful to better allocate resources and manage the patients' needs. The preventive measures put in place in Italy seemed to work, but this first experience with Covid-19 crisis highlighted the chronic problems of the Italian health system and the authors believe that all have to "learn the lesson" to be better prepared in the future
Greenhalgh et al., 2020 [18]	*Int J Clin Pract*	Retrospective	UK	The Covid-19 pandemic has had a profound effect of the provision of trauma and orthopaedic surgery. The authors report a significant decrease in all orthopaedic referrals during the pandemic, leading to a greatly reduced number of trauma operations performed. This has allowed for reallocation of staff and resources. The authors suggest that a plan for the lifting of social restrictions be made, since this lifting may lead to an increase in patients presenting with trauma requiring operative intervention
Gumina et al., 2020 [28]	*J Shoulder Elbow Surg*	Case Series	Italy	During the Covid-19 period, we provided a reduced number of health services, especially for patients with low-energy trauma and for those who underwent sports and traffic accidents. However, during the Covid-19 period, elderly subjects remain exposed to shoulder and elbow trauma due to low-energy (domestic) falls. The subsequent hospitalization of these patients has contributed to making it more difficult to manage the hospital wards that are partly occupied by Covid-19 patients
Gumina et al*.,* 2021 [25]	*JSES Int*	Retrospective	Italy	The pandemic forced us to become aware of the ways and places where skeletally immature subjects report shoulder and elbow traumas; therefore, it would be desirable that more considerable attention be directed toward the prevention of injury in areas at risk
Hernigou et al., 2020 [34]	*Int Orthop*	Retrospective Observational	Belgium	Staying home during the Covid-19 pandemic decreased trauma frequency of 32%. The structural organization in our hospital allowed us to reduce the time to surgery and ultimately hospital stay, thereby maximizing the already stretched medical resources available to treat all the patients who needed orthopedic care during this period
Luceri et al., 2020 [26]	*J Orthop Surg Res*	Retrospective	Italy	Social isolation certainly reduced the risk of trauma among the general population, and the fear of contagion probably kept non-urgent patients away from the emergency department. Evidence-based programs are fundamental to identify new strategies to maximize National Health System resources and decrease the time which patients spend in the emergency department, reducing overcrowding
Luengo-Alonso et al., 2020 [22]	*Int Orthop*	Single-Centre Cross-sectional	Spain	Detailed protocols should be standardized for surgical departments during the pandemic. This paper offers a general view in how this virus affects an orthopaedic unit and could serve as a protocol and example for orthopaedic and trauma units. Even in the worst scenario, an orthopaedic and trauma unit could offer an effective, efficient, and quality service. SARS-CoV-2 will set up a new paradigm for health care in orthopaedics and trauma
Maniscalco et al., 2020 [29]	*Acta Biomed*	Retrospective	Italy	In the first two months of the Italian epidemic, in the cities of Piacenza and Parma over 80% of deaths have occurred in patients over 70 years old. Even if preliminary, our study shows a significant increase in death in elderly patients surgically treated for proximal femur fractures, particularly in the Piacenza Hospital
Mitkovic et al., 2020 [33]	*Int Orthop*	Retrospective	Serbia	Restricted going outside the home for 54 days has the influence in total number of fractures and gender distribution in femoral neck fractures. The method of external fixation used could be assumed as a reducing factor of intraoperative virus pandemic propagation among medical staff
Murphy et al., 2020 [31]	*Injury*	Retrospective	UK	An association between the outbreak of the pandemic and a reduction in referral numbers to our department has been demonstrated. The direct cause of this may be multifactorial but proposing that it is, in part, due to the social distancing measures introduced by the government is certainly conceivable. The patterns of injury would reflect this also with low energy and fragility trauma persisting whilst injuries associated with younger people have reduced. We would suggest that information such as this could be useful in healthcare planning and resource allocation in future pandemic situations
Nunez et al., 2020 [16]	*Injury*	Retrospective Observational	Spain	While most traumatological presentations decreased in frequency over the course of the outbreak, the number of osteoporotic hip fractures remained stable. Thus, contingency plans in times of crisis need to be carefully targeted, and to keep in mind certain public health issues that do not decrease, despite a State of Emergency, like osteoporotic hip fractures
ORCA Collaboration, 2020 [20]	*Surgeon*	Survey-based	UK	We found a 97% reduction in elective operating, 64% reduction in elective outpatient activity and 37% reduction in operative trauma. 58% of trainees continued working in T&O clinics, with an average of 6 operative cases over this period. Our modelling suggests that the impact on training will persist; counter-measures must be incorporated into central recovery planning
Park et al., 2020 [21]	*Acta Orthop*	Longitudinal Observational	UK	The impact of the Covid-19 pandemic has led to a decline in the number of acute trauma referrals, admissions (but increased risk and odds ratio), operations, and aerosolizing anesthetic procedures since implementing social distancing and lockdown measures during the "golden month."
Peiro-Garcia et al., 2020 [24]	*J Child Orthop*	Retrospective Observational	Spain	According to our results, the pandemic has significantly affected our daily practice by decreasing elective surgeries and onsite clinics, but other activities have increased. As we have implemented telemedicine and new technologies to adapt to this setback, we should take advantage of the situation to change our practice in the future to better allocate our health resources and to anticipate outbreaks
Poggetti et al., 2020 [17]	*J Clin Orthop Trauma*	Retrospective	Italy	Even during drastic movement restrictions and the prolonged suspension of work and leisure activities secondary to Covid-19 epidemic in 2020, hand and wrist traumas rate remained almost the same compared to the same period of the previous year. Nevertheless, a significant change in the etiology and patient age was registered. In fact, sport and traffic-related traumas decreased respect to domestic traumas, while the previous prevalent involvement of young adults was surpassed by accidental hand traumas in the elderly and active adults
Ruggieri et al., 2020 [37]	*J Orthop Surg Res*	Retrospective	Italy	Extensive swab test of all people (even if asymptomatic) and proactive tracing and quarantining of potential Covid-19 positive patients may diminish the virus spread
Staunton et al., 2020 [36]	*Surgeon*	Retrospective	Ireland	The majority of trauma referred to our Dublin based centre during Covid-19 related population restrictions appears to be home based and trauma volumes have decreased. Significant reductions are apparent in work and sport related injuries suggestive of compliance with Covid-19 activity guidelines. Maintaining existing standards of treatment requires dedicated planning
Susgand et al., 2020 [19]	*Acta Orthop*	Retrospective	UK	The majority of trauma referred to our Dublin based centre during Covid-19 related population restrictions appears to be home based and trauma volumes have decreased. Significant reductions are apparent in work and sport related injuries suggestive of compliance with Covid-19 activity guidelines. Maintaining existing standards of treatment requires dedicated planning
Tamburelli et al., 2020 [39]	*J Orthop*	Retrospective	Italy	A 50% reduction of surgical procedures during the last three months was observed compared with the same period of time in 2019. The compliance with the containment rules for the spread of the infection, were sufficient to allow safe surgical activity for the medical teams and patients
von Dercks et al., 2020 [27]	*Orthopade*	Retrospective	Germany	The measures taken by the Government are an important pillar for the economic security of German hospitals. The lack of differentiation of measures by specialty leads to insufficient compensation for orthopaedics and trauma surgery
Zagra et al., 2020 [38]	*Int Orthop*	Retrospective	Italy	These numbers show the radical changed scenario in an orthopaedic center in Milan during Covid-19 pandemic. Elective surgery declined rapidly going close to zero, outpatient admissions were restricted to cases that cannot be postponed, while emergencies increased due to the role played by the hospital as referral orthopaedic centre during the pandemic. The still ongoing emergency will have important impacts on the overall orthopaedic healthcare management for the next months

Nine studies reported the impact of lockdown during the Covid-19 pandemic on injuries at level I trauma centres in Europe [40–48]. Compared to the pre-Covid-19 era, there was a significant reduction between 12.2% and 69.75% of patients presenting to trauma departments [40–48]. Three studies showed no significant reductions of major trauma, defined as an injury severity score (ISS) of greater than 15 [42, 44, 48]. In one study, significantly more polytrauma patients were reported during the Covid-19 period [42]. Road Traffic Collisions (RTCs), in the 2020 baseline time, accounted for 12.0 to 31.2% of trauma call activations. Conversely, in the period 2019, RTCs represented 14.0 to 54.5% of trauma call activations [40, 41, 43, 45–47]. The studies which reported data form European trauma registries during the year 2020 versus the pre-Covid-19 era are shown in Table 2.

**Table 2:** Studies which reported data from European hospital trauma registries during the year 2020 versus the pre-Covid-19 era

Author, year	Journal	Design	Country	Main findings
Adiamah et. Al, 2021 [41]	*Eur J Trauma Emerg Med*	Retrospective cohort	UK	During the SARS-CoV-2 pandemic and the associated national lockdown there was a significant reduction in number of trauma admissions. Patients admitted during the Covid-19 pandemic were older, frailer, more co-morbid and had an associated increased risk of mortality
Azbel et al., 2021 [44]	*BMC Emerg Med*	Retrospective cohort	Finland	The societal restrictions imposed by the Finnish government to curb the spread of Covid-19 had a significant effect on the number of EMS calls related to trauma in the capital area. The number of injured patients intoxicated by alcohol decreased significantly and the decrease was temporally related to the lockdown which included the closure of bars and nightclubs
Esteban et al. 2020 [45]	*Bone Join Open*	Retrospective	Spain	A marked drop in the total number of visits to our traumatology ED was observed, as well as a relative increase in major injury visits and a relative fall in the minor ones
Giudici et al., 2021 [46]	*World J Emerg Med*	Retrospective cohort	Italy	The emergency lockdown during the Covid-19 pandemic in Lombardia led to a reduction of major trauma, especially road-related injuries. The number of patients with intentional injuries admitted to the active level 1 trauma centers was greatly increased during the lockdown and this result would merit further analysis to assess the role of pre-existing factors and their interaction with the imposed restrictions. An increase in centralization to fewer facilities with high level of care obtained satisfactory results in the capability of the health system to take care of trauma emergencies while Covid-19 patients overwhelmed resources of most hospitals
Hakkenbrak et al., 2021 [42]	*Scand J Trauma Resusc Emerg Med*	Retrospective cohort	Netherlands	The overall in-hospital healthcare consumption was only marginally reduced and the number of surgically treated patients relatively increased. More severely injured patients and a higher percentage of patients in need for hospital admission were observed. Higher percentages of patients were treated surgically for extremity injuries. Results of this study can be used to optimize the use of hospital capacity and anticipate health care planning in future outbreaks for trauma patients
Helen et al., 2021 [47]	*Swiss Medical Weekly*	Retrospective cohort	Switzerland	In the first year of the Covid-19 pandemic, fewer patients with major trauma were admitted to the institution. However, the patients admitted were more severely injured and more often died within 30 days
Kreis et al., 2021 [43]	*Eur J Trauma Emerg Sur*	Retrospective cohort	Germany	This analysis shows a decrease of total patient numbers in an emergency department of a Level I trauma centre and a decrease of the total number of operations during the shutdown period. Furthermore, trauma mechanism changed with less traffic, work and sports related accidents
Moyer et al., 2021 [48]	*Scand J Trauma Resusc Emerg Med*	Retrospective cohort	France	During the Covid-19 pandemic period and more specifically during lockdown, the study demonstrated a 50% reduction in road traffic accidents with no increase in alternative injury mechanisms, such as assault or suicide. The in-hospital observed and predicted mortality and a number of crucial process indicators remained stable compared to previous years suggesting a sufficient resilience of the trauma networks assessed to absorb the spring 2020 pandemic hit. This study suggests that the care for major trauma patients was not substantially impacted by the SARS-CoV-2, 2020 first phase in France
Nia et al., 2021 [40]	*Wien Klin Wochenschr*	Retrospective	Austria	Although trauma of all age groups and severities will continue to occur, the tendency during a lockdown will be a greatly reduced case load. Nevertheless, with no significant drop in major injuries, resources need to remain readily available for any future waves The importance of versatility in managing limited resources has been highlighted, always adapting to an ever-changing situation. This will ensure the highest levels of service are maintained, reducing complications and ultimately improving patient outcomes

Team sports traumas evidenced a considerable reduction during the pandemic [49–51]. The injury rate remains similar in the German Bundesliga and Italian Serie A soccer leagues after the lockdown [52, 53]. Paediatric traumas decreased by 50% [54–56]. The impact of the pandemic on the incidence of fragility fractures is uncertain. Most studies found no difference in the rate of fragility femoral fractures compared to the same pre pandemic period [57–62]. Few studies reported a reduced trend of fractures compared to the pre pandemic period [63, 64]. Patients who experienced hip fragility fractures in the 2020 pandemic had a greater mortality compared to the same period of the pre pandemic era [65, 66]. The 30- and 90-day mortality in positive patients with fragility hip fractures was greater, as was the time span from injury to surgical treatment, and the hospitalisation [59, 67–71].

## Discussion

The Covid-19 pandemic impacted significantly the healthcare landscape worldwide, requiring considerable organizational efforts. According to the main findings of the present study, the overall trend of consultations, surgeries, and the rate of traumas and fragility fractures appear to decrease during the 2020 European COVID pandemic compared to the same period of the pre-pandemic era.

The participation in (team) sports activities was globally limited: several sporting events were suspended as a result of public safety restrictions. The shutdown periods affected training and competition in many sports activities, changing injury rates and patterns, with new implications for sports medicine [43]. However, no increased injury rate was observed in the German Bundesliga after the lockdown [52]. Accordingly, in the Italian Serie A soccer league a similar injury rate at 1000 game-hours in the pre- and post-lockdown period was found [53]. In contrast, when evaluating the impact of the Covid-19 lockdown on fitness in elite handball players, Fikenzer et al. [49] reported a reduced endurance capacity without team training despite a home-based strength and endurance program. The authors suggested a qualified supervision of individual home-based training programs to avoid the implementation of inadequate training concepts [49]. The effect of training restrictions due to Covid-19 associated emotional and physical stress was evaluated in national level Eventing horse-riding athletes [50]. The lockdown decreased performance outcomes of horse-riders in Eventing competitions [50]. Surprisingly, dressage was found to be the most affected discipline, when compared to cross-country and show-jumping. Faulkner et al. [51] evaluated cycling injuries in Scotland during the first Covid-19 lowdown period in a multi-centre study. The study group reported an uptake of cycling and a significant increase in the number of cycling related injuries requiring orthopaedic intervention, particularly with a greater proportion of female and elderly cyclists compared with similar time periods in 2018 and 2019 [51].

In paediatric sports traumatology, the concept of social distancing, school cancellations, and cessation of organized sports had a major impact on musculoskeletal injuries. Clavicula fractures were diagnosed more frequently compared to 2019. Bolzinger et al. [54] studied the epidemiology of paediatric injuries after the 8-week lockdown in France. The authors found an overall decrease of 50% in paediatric trauma, but an increased rate of domestic accidents (59% vs. 23%) and trampoline accidents (16% vs. 5%) [54]. Clos et al. [55] reported that serious sledding-related injuries increased significantly four- to five-fold in paediatric patients during the winter season of 2020–2021, whereas the number of snowboarding and skiing injuries decreased due to closed ski resorts [55]. Voth et al. [72] reported a rising trend of extremity fractures and sport injuries in children aged 8–12 years; however, their data refer to a prior period in 2018 and, therefore, do not include later effects of the Covid-19 pandemic. Darling et al. [56] analysed the effects of Covid-19 lockdowns on paediatric lower limb trauma. Throughout the lockdown periods, paediatric patients were younger (7 versus 11 years) and they were less likely to be injured as a result of sport [56]. Furthermore, the average rate of referrals and waiting time to receive surgical care dropped significantly. In this context, the role of telemedicine and telehealth is continuing to evolve for both side-line and clinical care of sport-related injuries [73–77]. While the scientific evidence is still evolving, various effects of the Covid-19 pandemic have affected both epidemiology and the clinical care for sports injuries.

The real impact of the COVID pandemic on the incidence of fragility fractures is uncertain. Being more common in outdoor activities, non-hip fragility fractures (e.g., forearm, upper arm, ankle, foot) may have been decreased [78–80]. On the other hand, the number of fragility hip fractures, which happen more frequently indoor, should be expected to have increased [81–83]. Current evidence is contradictory and within the same country a high variability is also common [84]. In a retrospective cohort study including overall 91,160 elderly people with hip fracture in France, hip fractures decreased by 11% compared to the pre pandemic period [63]. In another retrospective analysis of 236 patients following hip fracture, Ojeda-Thies et al. reported that the trend of hip fractures diminished by up to 26% compared to the previous year before the pandemic [64]. On the other hand, Ogliari et al. [57] evidenced no significant changes in the trend for fragility fractures in the United Kingdom with respect to the pre-pandemic period. In their study, the authors evaluated 6681 outpatients with non-hip fragility fractures and 1752 inpatients admitted for hip fracture [57]. Also Hampton et al. [58] found no difference in the rate of hip and non-hip fragility fractures during the 2020 pandemic compared to the same period of the previous year. Malik-Tabassum et al. [59] performed an observational, retrospective, multicentre study including 6 hospitals in the South East of England (767 patients). Compared to the same period one year before the pandemic, the authors found higher mortality in COVID positive patients, whereas no difference in the incidence of hip fractures was found [59]. The authors found that non-hip fractures were decreased, while there was no change in inpatient admissions for hip fractures [57]. Scott et al. [60] conducted a cohort study including 2876 patients who had been referred to the orthopaedic trauma service in Ireland. Femoral fragility fractures did not change significantly during the pandemic from the pre-pandemic period [60]. Nevertheless, the authors found a relative greater incidence of non-hip fragility fractures during the COVID-19 pandemic compared to the pre-pandemic period [60]. In a recent multicentre study [61] including 580 patients, no difference in the rate of femoral fractures was found alike. However, the authors evidenced a tendency to treat conservatively such fractures, along with a reduced hospitalisation and arthroplasties performed [61]. Similar findings were confirmed by Mazeda et al. [62] in a retrospective observational study involving 162 patients with a negative COVID PCR test. Another aspect to consider which may have an influence on the rate of fragility fractures, is the reduction of the routinely osteoporotic prevention cares. Indeed, the screening for osteoporosis dropped, with only 50% of performed bone density measurements (DXA) in comparison to the pre pandemic situation [85, 86]. DXA rates slowly increased to nearly 75% of pre-pandemic counts to the end of 2020 again [87]. Postoperative care also suffered during the pandemic. The incidence of pressure ulcers in patients following surgery for femoral fractures was 21%, a considerable increase when compared to the 10% of the pre pandemic era [88]. Overall, patients who experienced hip fragility fractures in the 2020 pandemic had a greater mortality compared to the same period of the pre-pandemic era [65, 66]. COVID infection directly contributed to increase the 30- and 90-day mortality following fragility hip fractures, not explained by patient characteristics [67–70]. Moreover, a longer time span from injury to surgical treatment and a longer hospitalisation was evidenced in comparison to the preceding year 2019, when the pandemic began [71].

If there are sufficient resources to treat current and potential future Covid-19 patients, elective surgery may be gradually resumed under a continuous monitoring of the infection rate. These resources include adequate intensive care units (ICU) and non-ICU departments, ventilators, personal protection equipment, and workforce projections to manage elective and emergency circumstances, and concurrently the capability to manage Covid-19 patients. If there are not enough resources, clinicians should consider the incubation curve of Covid-19. Previous evidence reported that the maximum estimated incubation for Covid-19 is up to 2 weeks, and 75% of cases develop symptoms within a week [89]. Given these assumptions, surgery should only be planned if there is a sustained reduction in the local infections rate for a period of at least 2 weeks [90, 91]. Moreover, given the viral shedding in infected individuals which ranged from 8 to 37 days (median 20) [92], patients who had a previous Covid-19 infection should be also retested within 6 weeks before the rescheduled surgery. Early diagnosis and isolation of positive patients and healthcare workers, and sufficient equipment resources are pivotal to prevent nosocomial transmission [93]. Specific infection rate thresholds should be set by the healthcare facilities to re-suspend surgeries [94]. As the prevalence of asymptomatic patients remains unknown [95], rapid testing 3 to 5 days prior to surgery should be set as standard [96–98]. The prioritization of orthopaedic and trauma surgical procedures is a multidisciplinary process which involves clinical and non-clinical personnel [99]. This process should follow a standardized decision-making protocol, an equitable and transparent framework to assure efficacy and prevent ethical dilemmas and moral injuries. The Medically Necessary Time-Sensitive (MeNTS) can be used as priority scoring system. Patients are prioritized based on procedure factors, disease factors and patient factors [100–102]. Procedure factors include surgical duration, hospitalization length, risk of postoperative ICU, total estimated blood loss, intubation chance, and surgical sites and team. Disease factors embrace the efficacy of conservative management and exposure risk, impact of treatment delay on the outcome, and difficulty or risk of surgery. Patient factors include age, lung and cardiovascular disease, diabetes, immunosuppression, influence symptoms, and contact with positive persons to Covid-19 in the past 2 weeks. For each of these factors a value from 1 to 5 based is assigned on both objective measures and perceived clinical probabilities. Lower values were associated with greater outcome, reduced risk of Covid-19 transmission to the healthcare team, and/or reduced hospital resource use during the pandemic [103, 104]. The MeNTS has been also applied with success for difficult decisions on prioritization of surgery in the orthopaedic and trauma surgeries during the pandemic [105]. During the process prioritization of surgical procedures, during the time elapsed since the originally scheduled surgical date, the patient status may have changed and needs to be reassessed prior to surgery. Laboratory and radiological assessment, comorbidities evaluation, symptoms and physical examination should be updated. To reduce the length of the hospitalization, the Enhanced Recovery After Surgery (ERAS) protocol has been introduced in the early 1990s [106–108]. The application of ERAS in the orthopaedic and trauma surgery promoted early mobilization, optimizing pain control avoiding the use of opioids, nausea and vomiting prophylaxis, amelioration of the nutritional and hydration status [109–111]. The purpose of the ERAS during pandemic is to reduce the risk of Covid-19 transmission and infection, to reduce crowding and improve patient turnover. In a recent meta-analysis involving 20,843 participants, ERAS reduced the incidence of postoperative complications and the 30-day mortality, though the readmission rate within 30 days did not show any statistically significant improvement [112]. One way to reduce crowding and patient turnover is to follow patients who had postponed their surgery by means of telemedicine, to ensure a continuous monitoring. Physicians should thereby be prepared to react to an impending breakdown. Telemedicine is defined as healthcare delivered from a remote location by computer and telecommunications technology replacing face to face modality [113]. Telemedicine is considered a safe and effective means to deliver healthcare. Patients appreciate its convenience due to reduced appointment delays and time off work as well as decreased travelling times and costs [114]. In comparison to other medical disciplines, telemedicine demonstrated limited evolution and application in orthopaedics and trauma surgery before the Covid-2019 pandemic [115, 116]. During the first Covid-19 pandemic telemedicine was applied to prevent assemblage and to guarantee access to medical cares. Telemedicine in orthopaedics and trauma surgery had mostly developed for arthroplasty, fracture management, and pre- and postoperative cares [117]. Several clinical studies investigated the application of telemedicine during COVID pandemic, with satisfying results [118–122].

This study has several limitations. Data on clinical evidence on consultations, surgeries, and traumas in the field of orthopaedic and trauma surgery in Europe during 2020 compared to the pre-pandemic era presented a wide range of variation. However, beside such variability in data presentation, the overall trend (increase or decrease) is relatively comparable among the studies. Such variability may arise from the different nature of the health care systems, different levels of care, and the different between- and within-countries heterogeneities in definitions, methodologies, diagnoses, and related management of injuries. Additionally, some between countries anti-COVID regulations allowed the institutions to pursue their surgical activity in a different fashion. These heterogeneities may lie behind the mentioned variability in data presentation, and infer negatively with the reliability of the conclusion of the present study. Therefore, data from the present study should be considered carefully.

## Conclusion

The overall trend of consultations, surgeries, and the rate of traumas and fragility fractures appear to decrease during the 2020 European COVID pandemic compared to the same period of the pre-pandemic era. The impact of COVID on morbidity and mortality in orthopaedic and trauma surgery is still unclear. Given the high heterogeneity in the clinical evidence, results from the present study should be considered carefully.

## Data Availability

The data underlying this article are available in the article and in its online supplementary material.

## References

[1] Barry M, Alotaibi M, Almohaya A, Aldrees A, AlHijji A, Althabit N, Alhasani S, Akkielah L, AlRajhi A, Nouh T, Temsah MH, Al-Tawfiq JA (2021). Factors associated with poor outcomes among hospitalized patients with COVID-19: experience from a MERS-CoV referral hospital. J Infect Public Health.

[2] Al-Dorzi HM, Aldawood AS, Almatrood A, Burrows V, Naidu B, Alchin JD, Alhumedi H, Tashkandi N, Al-Jahdali H, Hussain A, Al Harbi MK, Al Zaibag M, Bin Salih S, Al Shamrani MM, Alsaawi A, Arabi YM (2021). Managing critical care during COVID-19 pandemic: the experience of an ICU of a tertiary care hospital. J Infect Public Health.

[3] Atzrodt CL, Maknojia I, McCarthy RDP, Oldfield TM, Po J, Ta KTL, Stepp HE, Clements TP (2020). A Guide to COVID-19: a global pandemic caused by the novel coronavirus SARS-CoV-2. FEBS J.

[4] Harter M, Bremer D, Scherer M, von dem Knesebeck O, Koch-Gromus U (2020). Impact of COVID-19-pandemic on clinical care, work flows and staff at a university hospital: results of an interview-study at the UKE. Gesundheitswesen.

[5] Wong J, Goh QY, Tan Z, Lie SA, Tay YC, Ng SY, Soh CR (2020). Preparing for a COVID-19 pandemic: a review of operating room outbreak response measures in a large tertiary hospital in Singapore. Can J Anaesth.

[6] Prachand VN, Milner R, Angelos P, Posner MC, Fung JJ, Agrawal N, Jeevanandam V, Matthews JB (2020). Medically necessary, time-sensitive procedures: scoring system to ethically and efficiently manage resource scarcity and provider risk during the COVID-19 pandemic. J Am Coll Surg.

[7] American Enterprise Institute National coronavirus response: a road map to reopening. 2020. Available at https://www.aei.org/research-products/report/national-coronavirus-response-a-road-map-to-reopening/. Accessed in March 2021.

[8] Halawi MJ, Wang DD, Hunt TR (2020). What's important: weathering the COVID-19 crisis: time for leadership, vigilance, and unity. J Bone Joint Surg Am.

[9] At: https://www.cms.gov/files/document/covid-flexibility-reopen-essential-non-covid-services.pdf. Accessed in March 2021 CfMMSCfMMsCrR-oftpn-en-C-hpIA.

[10] Jensen RD, Bie M, Gundso AP, Schmid JM, Juelsgaard J, Gamborg ML, Mainz H, Rolfing JD (2020). Preparing an orthopedic department for COVID-19. Acta Orthop.

[11] Wang Y, Zeng L, Yao S, Zhu F, Liu C, Di Laura A, Henckel J, Shao Z, Hirschmann MT, Hart A, Guo X (2020). Recommendations of protective measures for orthopedic surgeons during COVID-19 pandemic. Knee Surg Sports Traumatol Arthrosc.

[12] Haddad FS (2020). COVID-19 and orthopaedic and trauma surgery. Bone Joint J.

[13] Howick J CI, Glasziou P, Greenhalgh T, Carl Heneghan, Liberati A, Moschetti I, Phillips B, Thornton H, Goddard O, Hodgkinson M. The 2011 Oxford CEBM levels of evidence. Oxford Centre for Evidence-Based Medicine. 2011. Available at http://www.cebm.net/index.aspx?o=5653

[14] Page MJ, McKenzie JE, Bossuyt PM, Boutron I, Hoffmann TC, Mulrow CD, Shamseer L, Tetzlaff JM, Akl EA, Brennan SE, Chou R, Glanville J, Grimshaw JM, Hrobjartsson A, Lalu MM, Li T, Loder EW, Mayo-Wilson E, McDonald S, McGuinness LA, Stewart LA, Thomas J, Tricco AC, Welch VA, Whiting P, Moher D (2021). The PRISMA 2020 statement: an updated guideline for reporting systematic reviews. BMJ.

[15] Benazzo F, Rossi SMP, Maniscalco P, Moretti B, Vaienti E, Ruggieri P, Masse A, Medici A, Formica A, Di Maggio B, Caiaffa V, Mosconi M, Murena L, D'Angelo F, Belluati A, Mazza EL, Rivera F, Castelli A, Ghiara M, Rosolani M, Cioffi R, Pezzella R, Scaravilli G, Bove G, Stissi P, Mazzacane M, Quattrini F, Ciatti C, Trovarelli G, Pala E, Angelini A, Sanna F, Nonne D, Colombelli A, Raggini F, Puzzo A, Canton G, Maritan G, Iuliano A, Randelli P, Solarino G, Moretti L, Vicenti G, Garofalo N, Nappi V, Ripanti S, Chinni C, Pogliacomi F, Visigalli A, Bini N, Aprato A, Perticarini L (2020). The orthopaedic and traumatology scenario during Covid-19 outbreak in Italy: chronicles of a silent war. Int Orthop.

[16] Nunez JH, Sallent A, Lakhani K, Guerra-Farfan E, Vidal N, Ekhtiari S, Minguell J (2020). Impact of the COVID-19 pandemic on an emergency traumatology service: experience at a tertiary trauma centre in Spain. Injury.

[17] Poggetti A, Del Chiaro A, Nucci AM, Suardi C, Pfanner S (2021). How hand and wrist trauma has changed during covid-19 emergency in Italy: incidence and distribution of acute injuries. What to learn?. J Clin Orthop Trauma.

[18] Greenhalgh M, Dupley L, Unsworth R, Boden R (2021). Where did all the trauma go? A rapid review of the demands on orthopaedic services at a UK Major Trauma Centre during the COVID-19 pandemic. Int J Clin Pract.

[19] Sugand K, Park C, Morgan C, Dyke R, Aframian A, Hulme A, Evans S, Sarraf KM, Baker C, Bennett-Brown K, Simon H, Bray E, Li L, Lee N, Pakroo N, Rahman K, Harrison A (2020). Impact of the COVID-19 pandemic on paediatric orthopaedic trauma workload in central London: a multi-centre longitudinal observational study over the "golden weeks". Acta Orthop.

[20] orca@eoeortho.com OCEa, Collaborative O. The response of Trauma and Orthopaedic Departments to the first four weeks of lockdown for the COVID-19 pandemic—a trainee-led analysis of the East of England. Surgeon. 2021;191:e14–e1910.1016/j.surge.2020.07.007 10.1016/j.surge.2020.07.007 PMC7395609 32830040

[21] Park C, Sugand K, Nathwani D, Bhattacharya R, Sarraf KM (2020). Impact of the COVID-19 pandemic on orthopedic trauma workload in a London level 1 trauma center: the "golden month". Acta Orthop.

[22] Luengo-Alonso G, Perez-Tabernero FG, Tovar-Bazaga M, Arguello-Cuenca JM, Calvo E (2020). Critical adjustments in a department of orthopaedics through the COVID-19 pandemic. Int Orthop.

[23] Giuntoli M, Bonicoli E, Bugelli G, Valesini M, Manca M, Scaglione M (2020). Lessons learnt from COVID 19: an Italian multicentric epidemiological study of orthopaedic and trauma services. J Clin Orthop Trauma.

[24] Peiro-Garcia A, Corominas L, Coelho A, DeSena-DeCabo L, Torner-Rubies F, Fontecha CG (2020). How the COVID-19 pandemic is affecting paediatric orthopaedics practice: a preliminary report. J Child Orthop.

[25] Gumina S, Proietti R, Villani C, Carbone S, Candela V (2021). The impact of COVID-19 on shoulder and elbow trauma in a skeletally immature population: an Italian survey. JSES Int.

[26] Luceri F, Morelli I, Accetta R, Mangiavini L, Maffulli N, Peretti GM (2020). Italy and COVID-19: the changing patient flow in an orthopedic trauma center emergency department. J Orthop Surg Res.

[27] von Dercks N, Korner C, Heyde CE, Theopold J (2020). How badly is the coronavirus pandemic affecting orthopaedic and trauma surgery clinics? An analysis of the first 5 weeks. Orthopade.

[28] Gumina S, Proietti R, Polizzotti G, Carbone S, Candela V (2020). The impact of COVID-19 on shoulder and elbow trauma: an Italian survey. J Shoulder Elbow Surg.

[29] Maniscalco P, Poggiali E, Quattrini F, Ciatti C, Magnacavallo A, Vercelli A, Domenichini M, Vaienti E, Pogliacomi F, Ceccarelli F (2020). Proximal femur fractures in COVID-19 emergency: the experience of two Orthopedics and Traumatology Departments in the first eight weeks of the Italian epidemic. Acta Biomed.

[30] Druel T, Andeol Q, Rongieras F, Bertani A, Bordes M, Alvernhe A (2020). Evaluation of containment measures' effect on orthopaedic trauma surgery during the COVID-19 pandemic: a retrospective comparison between 2019 and 2020. Int Orthop.

[31] Murphy T, Akehurst H, Mutimer J (2020). Impact of the 2020 COVID-19 pandemic on the workload of the orthopaedic service in a busy UK district general hospital. Injury.

[32] Ghermandi R, Pipola V, Terzi S, Tedesco G, Cavallari C, Bandiera S, Barbanti Brodano G, Evangelisti G, Girolami M, Gasbarrini A (2020). The impact of SARS-CoV-2 pandemic on oncologic and degenerative spine surgery department activity: the experience of Rizzoli Orthopaedic Institute under COVID-19 lockdown. Eur Rev Med Pharmacol Sci.

[33] Mitkovic MM, Bumbasirevic M, Milenkovic S, Gajdobranski D, Bumbasirevic V, Mitkovic MB (2021). Influence of coronavirus disease 2019 pandemic state of emergency in orthopaedic fracture surgical treatment. Int Orthop.

[34] Hernigou J, Morel X, Callewier A, Bath O, Hernigou P (2020). Staying home during “COVID-19” decreased fractures, but trauma did not quarantine in one hundred and twelve adults and twenty eight children and the “tsunami of recommendations” could not lockdown twelve elective operations. Int Orthop.

[35] Andreata M, Faraldi M, Bucci E, Lombardi G, Zagra L (2020). Operating room efficiency and timing during coronavirus disease 2019 outbreak in a referral orthopaedic hospital in Northern Italy. Int Orthop.

[36] Staunton P, Gibbons JP, Keogh P, Curtin P, Cashman JP, O'Byrne JM (2021). Regional trauma patterns during the COVID-19 pandemic. Surgeon.

[37] Ruggieri P, Trovarelli G, Angelini A, Pala E, Berizzi A, Donato D (2020). COVID-19 strategy in organizing and planning orthopedic surgery in a major orthopedic referral center in an area of Italy severely affected by the pandemic: experience of the Department of Orthopedics, University of Padova. J Orthop Surg Res.

[38] Zagra L, Faraldi M, Pregliasco F, Vinci A, Lombardi G, Ottaiano I, Accetta R, Perazzo P, D'Apolito R (2020). Changes of clinical activities in an orthopaedic institute in North Italy during the spread of COVID-19 pandemic: a seven-week observational analysis. Int Orthop.

[39] Tamburrelli FC, Meluzio MC, Perna A, Santagada DA, Genitiempo M, Zirio G, Proietti L (2020). Spinal surgery in COVID-19 pandemic era: One trauma hub center experience in central-southern Italy. J Orthop.

[40] Nia A, Popp D, Diendorfer C, Apprich S, Munteanu A, Hajdu S, Widhalm HK (2021). Impact of lockdown during the COVID-19 pandemic on number of patients and patterns of injuries at a level I trauma center. Wien Klin Wochenschr.

[41] Adiamah A, Thompson A, Lewis-Lloyd C, Dickson E, Blackburn L, Moody N, Gida S, La Valle A, Reilly JJ, Saunders J, Brooks A, Group ITS. The ICON Trauma Study: the impact of the COVID-19 lockdown on major trauma workload in the UK. Eur J Trauma Emerg Surg. 2021;47(3):637–64510.1007/s00068-020-01593-w 10.1007/s00068-020-01593-w PMC7871318 33559697

[42] Hakkenbrak NAG, Loggers SAI, Lubbers E, de Geus J, van Wonderen SF, Berkeveld E, Mikdad S, Giannakopoulos GF, Ponsen KJ, Bloemers FW, group CO-tc (2021). Trauma care during the COVID-19 pandemic in the Netherlands: a level 1 trauma multicenter cohort study. Scand J Trauma Resusc Emerg Med.

[43] Kreis CA, Ortmann B, Freistuehler M, Hartensuer R, Van Aken H, Raschke MJ, Schliemann B (2021). Impact of the first COVID-19 shutdown on patient volumes and surgical procedures of a Level I trauma center. Eur J Trauma Emerg Surg.

[44] Azbel M, Heinanen M, Laaperi M, Kuisma M (2021). Effects of the COVID-19 pandemic on trauma-related emergency medical service calls: a retrospective cohort study. BMC Emerg Med.

[45] Esteban PL, Querolt Coll J, Xicola Martinez M, Cami Biayna J, Delgado-Flores L (2020). Has COVID-19 affected the number and severity of visits to a traumatology emergency department?. Bone Jt Open.

[46] Giudici R, Lancioni A, Gay H, Bassi G, Chiara O, Mare C, Latronico N, Pesenti A, Faccincani R, Cabrini L, Fumagalli R, Chieregato A, Briani L, Sammartano F, Sechi G, Zoli A, Pagliosa A, Foti G, Borotto E, Palo A, Valoti O, Botteri M, Carlucci M, Reitano E, Bini R (2021). Impact of the COVID-19 outbreak on severe trauma trends and healthcare system reassessment in Lombardia, Italy: an analysis from the regional trauma registry. World J Emerg Surg.

[47] Anwander H, Klingberg K, Gerber J, Bednarski P, Exadaktylos A, Muller M (2021). Major trauma during COVID-19 in a level 1 trauma centre in Switzerland—a cohort study comparing the years 2020 and 2019. Swiss Med Wkly.

[48] Moyer JD, James A, Gakuba C, Boutonnet M, Angles E, Rozenberg E, Bardon J, Clavier T, Legros V, Werner M, Mathais Q, Ramonda V, Le Minh P, Berthelot Y, Colas C, Pottecher J, Gauss T, The Traumabase G (2021). Impact of the SARS-COV-2 outbreak on epidemiology and management of major traumain France: a registry-based study (the COVITRAUMA study). Scand J Trauma Resusc Emerg Med.

[49] Fikenzer S, Fikenzer K, Laufs U, Falz R, Pietrek H, Hepp P (2021). Impact of COVID-19 lockdown on endurance capacity of elite handball players. J Sports Med Phys Fitness.

[50] Demarie S, Galvani C, Billat VL (2020). Horse-riding competitions pre and post COVID-19: effect of anxiety, sRPE and HR on performance in eventing. Int J Environ Res Public Health.

[51] Faulkner A, MacDonald DRW, Neilly DW, Davies PSE, Ha TT, Stevenson IM, Jariwala AC (2021). Cycling injuries requiring orthopaedic intervention during the first COVID-19 lockdown period: a multi-centre SCottish Orthopaedic Research collaborativE (SCORE) study. Surgeon.

[52] Krutsch W, Hadji A, Tröß T, Szymski D, Aus der Fünten K, Gärtner B, Alt V, Meyer T (2021). No increased injury incidence in the German Bundesliga after the SARS-CoV-2 virus lockdown. Arch Orthopaed Trauma Surg.

[53] Marotta N, Gimigliano A, Demeco A, Moggio L, Vescio A, Iona T, Ammendolia A. Impact of COVID-19 lockdown on the epidemiology of soccer muscle injuries in Italian Serie A professional football players. J Sports Med Phys Fitness.2021.10.23736/s0022-4707.21.12903-2 10.23736/S0022-4707.21.12903-2 34546026

[54] Bolzinger M, Lopin G, Accadbled F, Sales de Gauzy J, Compagnon R (2021). Pediatric traumatology in "green zone" during Covid-19 lockdown: a single-center study. Orthop Traumatol Surg Res.

[55] Clos M, Evain JN, Wroblewski I, Bouzat P, Mortamet G (2021). Serious sledding injuries in children dramatically increased during the COVID-19 pandemic. Acta Paediatr.

[56] Darling J, Nowicka M, Niazi N, Pillai A (2021). The effect of COVID-19 lockdowns on paediatric lower limb orthopaedic presentations. Arch Orthopaed Trauma Surg.

[57] Ogliari G, Lunt E, Ong T, Marshall L, Sahota O (2020). The impact of lockdown during the COVID-19 pandemic on osteoporotic fragility fractures: an observational study. Arch Osteoporos.

[58] Hampton M, Clark M, Baxter I, Stevens R, Flatt E, Murray J, Wembridge K (2020). The effects of a UK lockdown on orthopaedic trauma admissions and surgical cases: a multicentre comparative study. Bone Jt Open.

[59] Malik-Tabassum K, Robertson A, Tadros BJ, Chan G, Crooks M, Buckle C, Rogers B, Selmon G, Arealis G (2021). The effect of the COVID-19 lockdown on the epidemiology of hip fractures in the elderly: a multicentre cohort study. Ann R Coll Surg Engl.

[60] Scott CEH, Holland G, Powell-Bowns MFR, Brennan CM, Gillespie M, Mackenzie SP, Clement ND, Amin AK, White TO, Duckworth AD (2020). Population mobility and adult orthopaedic trauma services during the COVID-19 pandemic: fragility fracture provision remains a priority. Bone Jt Open.

[61] Wignall A, Giannoudis V, De C, Jimenez A, Sturdee S, Nisar S, Pandit H, Gulati A, Palan J (2021). The impact of COVID-19 on the management and outcomes of patients with proximal femoral fractures: a multi-centre study of 580 patients. J Orthop Surg Res.

[62] Mazeda C, Santos PB, Vilas-Boas P, Antao J, Barcelos A (2021). What happened to hip fragility fractures during COVID-19 pandemic?. Acta Reumatol Port.

[63] Paccou J, Lenne X, Ficheur G, Theis D, Cortet B, Bruandet A (2021). Analysis of hip fractures in france during the First COVID-19 lockdown in spring 2020. JAMA Netw Open.

[64] Ojeda-Thies C, Cuarental-Garcia J, Ramos-Pascua LR (2021). Decreased volume of hip fractures observed during COVID-19 lockdown. Eur Geriatr Med.

[65] Orfanos G, Al Kaisi K, Jaiswal A, Lim J, Youssef B (2021). The effect of COVID-19 pandemic on the care of fragility hip fracture patients in the United Kingdom. A case control study in a major trauma centre. Surgeon.

[66] Hall AJ, Clement ND, Farrow L, MacLullich AMJ, Dall GF, Scott CEH, Jenkins PJ, White TO, Duckworth AD, Group IM-SS. IMPACT-Scot report on COVID-19 and hip fractures. Bone Joint J. 2020;102-B(9):1219–1228.10.1302/0301-620X.102B9.BJJ-2020-1100.R1 10.1302/0301-620X.102B9.BJJ-2020-1100.R1 32634029

[67] Crozier-Shaw G, Hughes AJ, Conlon B, Sheehan E, Merghani K (2021). Hip fracture care during Covid-19: a regional trauma centre's experience. Ir J Med Sci.

[68] Mackay ND, Wilding CP, Langley CR, Young J (2020). The impact of COVID-19 on trauma and orthopaedic patients requiring surgery during the peak of the pandemic: a retrospective cohort study. Bone Jt Open.

[69] Alcock H, Moppett EA, Moppett IK (2021). Early mortality outcomes of patients with fragility hip fracture and concurrent SARS-CoV-2 infection : a systematic review and meta-analysis. Bone Jt Open.

[70] Zamora T, Sandoval F, Demandes H, Serrano J, Gonzalez J, Lira MJ, Klaber I, Carmona M, Botello E, Schweitzer D (2021). Hip fractures in the elderly during the COVID-19 pandemic: a Latin-American perspective with a minimum 90-day follow-up. Geriatr Orthop Surg Rehabil.

[71] Ojeda-Thies C, Cuarental-Garcia J, Garcia-Gomez E, Salazar-Zamorano CH, Alberti-Marono J, Ramos-Pascua LR (2021). Hip fracture care and mortality among patients treated in dedicated COVID-19 and non-COVID-19 circuits. Eur Geriatr Med.

[72] Voth M, Sommer K, Schindler C, Frank J, Marzi I (2021). Rise of extremity fractures and sport accidents in children at 8–12 years and increase of admittance via the resuscitation room over a decade. Eur J Trauma Emerg Surg.

[73] Tam A, Plotsker E, Kim M, Thaller SR (2021). Telemedicine for sports-related injuries. J Craniofac Surg.

[74] Subramanyam V, Day MA, Kinderknecht JJ (2021). The role of telehealth in sideline management of sports-related injuries. Hss j.

[75] Womble MN, Reynolds E, Kissinger-Knox A, Collins MW, Kontos AP, West RV, Eagle S, Elbin RJ (2021). The emerging role of telehealth for concussion clinical care during the coronavirus (COVID-19) pandemic. J Head Trauma Rehabil.

[76] Scherer J, Back DA, Thienemann F, Kaufmann E, Neuhaus V, Willy C, Hepp P, Pape HC, Osterhoff G (2021). The effect of Covid-19 on the willingness to use video consultations among orthopedic and trauma outpatients: a multi-center survey in 1400 outpatients. Eur J Trauma Emerg Surg.

[77] Hertling S, Loos FM, Graul I (2021). Telemedicine as a therapeutic option in sports medicine: results of a nationwide cross-sectional study among physicians and patients in Germany. Int J Environ Res Public Health.

[78] Kelsey JL, Procter-Gray E, Hannan MT, Li W (2012). Heterogeneity of falls among older adults: implications for public health prevention. Am J Public Health.

[79] Kelsey JL, Berry SD, Procter-Gray E, Quach L, Nguyen US, Li W, Kiel DP, Lipsitz LA, Hannan MT (2010). Indoor and outdoor falls in older adults are different: the maintenance of balance, independent living, intellect, and Zest in the Elderly of Boston Study. J Am Geriatr Soc.

[80] Li W, Keegan TH, Sternfeld B, Sidney S, Quesenberry CP, Kelsey JL (2006). Outdoor falls among middle-aged and older adults: a neglected public health problem. Am J Public Health.

[81] Costa AG, Wyman A, Siris ES, Watts NB, Silverman S, Saag KG, Roux C, Rossini M, Pfeilschifter J, Nieves JW, Netelenbos JC, March L, LaCroix AZ, Hooven FH, Greenspan SL, Gehlbach SH, Diez-Perez A, Cooper C, Compston JE, Chapurlat RD, Boonen S, Anderson FA, Adachi JD, Adami S (2013). When, where and how osteoporosis-associated fractures occur: an analysis from the Global Longitudinal Study of Osteoporosis in Women (GLOW). PLoS One.

[82] Emaus N, Olsen LR, Ahmed LA, Balteskard L, Jacobsen BK, Magnus T, Ytterstad B (2011). Hip fractures in a city in Northern Norway over 15 years: time trends, seasonal variation and mortality: the Harstad Injury Prevention Study. Osteoporos Int.

[83] Nevitt MC, Cummings SR (1993). Type of fall and risk of hip and wrist fractures: the study of osteoporotic fractures. The study of osteoporotic fractures research group. J Am Geriatr Soc.

[84] Golinelli D, Lenzi J, Adorno E, Gianino MM, Fantini MP (2021). COVID-19 and regional differences in the timeliness of hip-fracture surgery: an interrupted time-series analysis. PeerJ.

[85] Fuggle NR, Singer A, Gill C, Patel A, Medeiros A, Mlotek AS, Pierroz DD, Halbout P, Harvey NC, Reginster JY, Cooper C, Greenspan SL (2021). How has COVID-19 affected the treatment of osteoporosis? An IOF-NOF-ESCEO global survey. Osteoporos Int.

[86] Peeters JJM, van den Berg P, van den Bergh JP, Emmelot-Vonk MH, de Klerk G, Lems WF, Winter EM, Zillikens MC, Appelman-Dijkstra NM (2021). Osteoporosis care during the COVID-19 pandemic in the Netherlands: a national survey. Arch Osteoporos.

[87] Cromer SJ, Yu EW (2021). Challenges and opportunities for osteoporosis care during the COVID-19 pandemic. J Clin Endocrinol Metab.

[88] Morri M, Forni C, Evangelista A, Broggian T, Ambrosi E, Orlandi AM (2021). The impact of the second wave of COVID-19 on outcomes in hip fracture patients: an observational study. Appl Sci.

[89] Lauer SA, Grantz KH, Bi Q, Jones FK, Zheng Q, Meredith HR, Azman AS, Reich NG, Lessler J (2020). The incubation period of coronavirus disease 2019 (COVID-19) from publicly reported confirmed cases: estimation and application. Ann Intern Med.

[90] Reopening AEINcrarmt. 2020. https://www.aei.org/research-products/report/national-coronavirus-response-a-road-map-to-reopening/. Accessed August 2021

[91] American Society of Anesthesiologists American College of Surgeons AopRN. American Hospital Association Joint statement: roadmap for resuming elective surgery after COVID-19 pandemic. 2020. https://www.asahq.org/about-asa/newsroom/news-releases/2020/04/joint-statement-on-elective-surgery-after-covid-19-pandemic Accessed March 2021

[92] Zhou F, Yu T, Du R, Fan G, Liu Y, Liu Z, Xiang J, Wang Y, Song B, Gu X, Guan L, Wei Y, Li H, Wu X, Xu J, Tu S, Zhang Y, Chen H, Cao B (2020). Clinical course and risk factors for mortality of adult inpatients with COVID-19 in Wuhan, China: a retrospective cohort study. Lancet.

[93] Pung R, Chiew CJ, Young BE, Chin S, Chen MI, Clapham HE, Cook AR, Maurer-Stroh S, Toh M, Poh C, Low M, Lum J, Koh VTJ, Mak TM, Cui L, Lin R, Heng D, Leo YS, Lye DC, Lee VJM, Singapore Novel Coronavirus Outbreak Research T. Investigation of three clusters of COVID-19 in Singapore: implications for surveillance and response measures. Lancet. 2020;395(10229):1039–1046.10.1016/S0140-6736(20)30528-6 10.1016/S0140-6736(20)30528-6 PMC7269710 32192580

[94] Facilities CfDCaPEppePnfh. https://www.cdc.gov/vhf/ebola/healthcare-us/ppe/calculator.html. Accessed March 2021

[95] Du Z, Xu X, Wu Y, Wang L, Cowling BJ, Meyers LA (2020). Serial interval of COVID-19 among publicly reported confirmed cases. Emerg Infect Dis.

[96] Chan JF, Yip CC, To KK, Tang TH, Wong SC, Leung KH, Fung AY, Ng AC, Zou Z, Tsoi HW, Choi GK, Tam AR, Cheng VC, Chan KH, Tsang OT, Yuen KY. Improved molecular diagnosis of COVID-19 by the novel, highly sensitive and specific COVID-19-RdRp/Hel real-time reverse transcription-PCR assay validated in vitro and with clinical specimens. J Clin Microbiol. 2020. 10.1128/JCM.00310-20 10.1128/JCM.00310-20 PMC7180250 32132196

[97] Hadaya J, Schumm M, Livingston EH (2020). Testing individuals for coronavirus disease 2019 (COVID-19). JAMA.

[98] Chew MH, Tan WJ, Ng CY, Ng KH (2020). Deeply reconsidering elective surgery: worldwide concerns regarding colorectal surgery in a COVID-19 pandemic and a Singapore perspective. Singapore Med J.

[99] Dyer GSM, Harris MB (2020). What's important: facing fear in the time of COVID-19. J Bone Joint Surg Am.

[100] Dincer MB, Guler MM, Gok AFK, Ilhan M, Orhan-Sungur M, Ozkan-Seyhan T, Koltka AK (2021). Evaluation of postoperative complication with medically necessary, time-sensitive scoring system during acute COVID-19 pandemic: a prospective observational study. J Am Coll Surg.

[101] Ermer JP, Ballester JMS, Go BC, Philipson B, Gabriel PE, Pryma DA, Fraker DL, Kelz RR, Wachtel H (2021). Endocrine surgical procedures during COVID-19: patient prioritization and time to surgery. J Surg Res.

[102] Sharma A, Matos S, Ettema SL, Gregory SR, Javadi P, Johnson MD, Stack BC, Jr., Crosby DL. Development and assessment of an otolaryngology-specific surgical priority scoring system. OTO Open. 2021;5(2):2473974X211012664. 10.1177/2473974X211012664 10.1177/2473974X211012664 PMC8114268 34017936

[103] Saleeby E, Acree R, Wieslander C, Truong C, Garcia L, Eckhardt S, Hari A, Al-Marayati L, Greenwell L, Holschneider CH (2021). Prioritizing surgical services during on-going pandemic response: modification and reliability of the medically necessary time sensitive surgery (MeNTS) scoring tool. J Med Syst.

[104] Marfori CQ, Klebanoff JS, Wu CZ, Barnes WA, Carter-Brooks CM, Amdur RL (2021). Reliability and validity of 2 surgical prioritization systems for reinstating nonemergent benign gynecologic surgery during the COVID-19 pandemic. J Minim Invasive Gynecol.

[105] Prabhakar SM, Decruz J, Kunnasegaran R (2021). The MeNT-OS score for orthopaedic surgery: an objective scoring system for prioritisation of orthopaedic elective surgeries during a pandemic. Indian J Orthop.

[106] Wei B, Tang C, Li X, Lin R, Han L, Zheng S, Xu Y, Yao Q, Wang L (2021). Enhanced recovery after surgery protocols in total knee arthroplasty via midvastus approach: a randomized controlled trial. BMC Musculoskelet Disord.

[107] Tang Z, Lu M, Qu C, Zhang Y, Li L, Li S, Qi L, Cheng C, Tian H (2021). Enhanced recovery after surgery improves short-term outcomes in patients undergoing esophagectomy. Ann Thorac Surg.

[108] Marulanda K, Purcell LN, Strassle PD, McCauley CJ, Mangat SA, Chaumont N, Sadiq TS, McNaull PP, Lupa MC, Hayes AA, Phillips MR (2021). A comparison of adult and pediatric enhanced recovery after surgery pathways: a move for standardization. J Surg Res.

[109] Pitter FT, Jorgensen CC, Lindberg-Larsen M, Kehlet H, Lundbeck Foundation Center for Fast-track H, Knee Replacement Collaborative G (2016). Postoperative morbidity and discharge destinations after fast-track hip and knee arthroplasty in patients older than 85 years. Anesth Analg.

[110] Quack V, Ippendorf AV, Betsch M, Schenker H, Nebelung S, Rath B, Tingart M, Luring C (2015). Multidisciplinary rehabilitation and fast-track rehabilitation after knee replacement: faster, better, cheaper? A survey and systematic review of literature. Rehabilit (Stuttg).

[111] Sibia US, MacDonald JH, King PJ (2016). Predictors of hospital length of stay in an enhanced recovery after surgery program for primary total hip arthroplasty. J Arthroplasty.

[112] Hu ZC, He LJ, Chen D, Li XB, Feng ZH, Fu CW, Xuan JW, Ni WF, Wu AM (2019). An enhanced recovery after surgery program in orthopedic surgery: a systematic review and meta-analysis. J Orthop Surg Res.

[113] Bashshur RL (1995). On the definition and evaluation of telemedicine. Telemed J.

[114] Kruse CS, Krowski N, Rodriguez B, Tran L, Vela J, Brooks M (2017). Telehealth and patient satisfaction: a systematic review and narrative analysis. BMJ Open.

[115] Parisien RL, Shin M, Constant M, Saltzman BM, Li X, Levine WN, Trofa DP (2020). Telehealth utilization in response to the novel coronavirus (COVID-19) pandemic in orthopaedic surgery. J Am Acad Orthop Surg.

[116] Buvik A, Bugge E, Knutsen G, Smabrekke A, Wilsgaard T (2016). Quality of care for remote orthopaedic consultations using telemedicine: a randomised controlled trial. BMC Health Serv Res.

[117] Behmanesh A, Sadoughi F, Mazhar FN, Joghataei MT, Yazdani S (2020). Tele-orthopaedics: a systematic mapping study. J Telemed Telecare.

[118] Ortiz-Pina M, Molina-Garcia P, Femia P, Ashe MC, Martin-Martin L, Salazar-Gravan S, Salas-Farina Z, Prieto-Moreno R, Castellote-Caballero Y, Estevez-Lopez F, Ariza-Vega P (2021). Effects of tele-rehabilitation compared with home-based in-person rehabilitation for older adult's function after hip fracture. Int J Environ Res Public Health.

[119] Claassen A, Schers HJ, Busch V, Heesterbeek PJC, van den Hoogen FHJ, Vliet Vlieland TPM, van den Ende CHM (2020). Preparing for an orthopedic consultation using an eHealth tool: a randomized controlled trial in patients with hip and knee osteoarthritis. BMC Med Inform Decis Mak.

[120] Ariza-Vega P, Castillo-Perez H, Ortiz-Pina M, Ziden L, Palomino-Vidal J, Ashe MC (2021). The journey of recovery: caregivers' perspectives from a hip fracture telerehabilitation clinical trial. Phys Ther.

[121] Pronk Y, Peters M, Sheombar A, Brinkman JM (2020). Effectiveness of a mobile eHealth app in guiding patients in pain control and opiate use after total knee replacement: randomized controlled trial. JMIR Mhealth Uhealth.

[122] Teot L, Geri C, Lano J, Cabrol M, Linet C, Mercier G (2020). Complex wound healing outcomes for outpatients receiving care via telemedicine, home health, or wound clinic: a randomized controlled trial. Int J Low Extrem Wounds.

